# The relationship between age, spirituality and general beliefs about how the world functions in cardiovascular patients

**DOI:** 10.1192/j.eurpsy.2026.11053

**Published:** 2026-07-31

**Authors:** O. V. Nikolaeva, L. V. Tarasova, E. L. Nikolaev, Y. V. Tsyganova, T. E. Nikolaeva, H. Kumar

**Affiliations:** 1 Department of Hospital Therapy; 2 Department of Social and Clinical Psychology; 3 Medical Faculty, I.N. Ulianov Chuvash State University, Cheboksary, Russian Federation

## Abstract

**Introduction:**

A person’s social perception of how the world actually functions can change with age. Illness can eventually lead a person to the review of their attitude to themselves, to the world and other people, of their moral values and will for self-perfection.

**Objectives:**

Our objective was to define the relationship between age, spirituality and general beliefs about how the world functions in cardiovascular patients of different sex and age.

**Methods:**

We polled 50 patients of both sexes aged from 33 to 76, who were undergoing inpatient treatment for cardiovascular diseases (CVD). We used a Russian Version of the Social Axioms Questionnaire by M. Bond and K. Leung (Leung K, Bond MH et al., 2002) made by A. Tatarko and N. Lebedeva (2020), and the Spiritual Personality Questionnaire by G. Ozhiganova (2019). The received data were statistically processed. We defined the presence of credible interrelations between the parameters with the help of correlation analysis at the significance level of p<.05.

**Results:**

In the course of the survey, we found out that the mean values of spirituality in CVD patients correspond to those of healthy people. The patients’ age is not interrelated with the level of their spirituality, but it correlates with general beliefs about how the world functions (social axioms). The older a patient is, the more frequently they have an ischemic heart disease (ICD) (r=-.43; p<.05), the more expressed are their social cynicism (r=.30; p<.05) and belief in their ability to control their own fate (r=.30; p<.05). ICD patients are more likely to believe it is possible to control their fate (r=.41; p<.05) and they are less flexible when solving their problems (r=-.34; p<.05). Psychological manifestations of spirituality in CVD patients are differently connected with social axioms. Thus, high morality and wisdom positively correlates with their perception of an award for efforts (r=.44; p<.05) and social complexity (r=.35; p<.05); reliability and responsibility – with religiousness (r=.35; p<.05), an award for efforts (r=.36; p<.05) and social complexity (r=.31; p<.05); spirituality of their relations – with an award for efforts (r=.57; p<.05) and social complexity (r=.51; p<.05); truthfulness and satisfaction – with religiousness (r=.41; p<.05), an award for efforts (r=.37; p<.05) and social complexity (r=.36; p<.05). The given research did not reveal any sex differences.

**Conclusions:**

We can characterize the surveyed CVD patients as having a standard level of spirituality, which has no connection with their age. As years go by, they can more negatively assess a human nature; however, they believe in the possibility of influencing the predetermination of fate. Their higher level of spirituality manifests itself in growing religiousness, expectation of awards for efforts and understanding of social complexity of the world.

**Disclosure of Interest:**

None Declared

